# A Preemptive Priority-Based Data Fragmentation Scheme for Heterogeneous Traffic in Wireless Sensor Networks

**DOI:** 10.3390/s18124473

**Published:** 2018-12-17

**Authors:** Anwar Ahmed Khan, Sayeed Ghani, Shama Siddiqui

**Affiliations:** 1 Faculty of Computer Science, Institute of Business Administration, Karachi 74400, Pakistan; sghani@iba.edu.pk; 2 Department of Computer Science, DHA Suffa University, Karachi 75500, Pakistan; shamasid@hotmail.com

**Keywords:** FROG-MAC, fragmentation, heterogeneous, IoT, priority

## Abstract

Prioritizing the heterogeneous traffic for Wireless Sensor Networks (WSNs) imposes an important performance challenge for Internet of Things (IoT) applications. Most past preemptive MAC schemes are based on scheduling the high priority packets earlier than those of lower priority. However, in a majority of these schemes, high priority traffic must wait for the ongoing transmission of lower priority traffic due to the non-availability of an interruption mechanism. This paper presents the design and high-level implementation details of a fragmentation scheme (FROG-MAC) for heterogeneous traffic in WSN. FROG-MAC aims at guaranteeing quick transmission of high priority/emergency traffic by interrupting ongoing on channel transmissions. High level implementation of FROG-MAC has been developed in MATLAB as a proof of concept. Traffic of two priorities was generated and a single hop star topology of 100 nodes was used for the experiments. Effect of the proposed fragmentation scheme has been evaluated on delay and Packet Drop Ratio (PDR) for both traffic types, by varying the packet size and fragment size. Simulation results have suggested that with the increasing packet size, the delay and PDR increase for both traffic types. When fragmentation was applied, the performance of high priority traffic significantly improved as compared to the low priority for both the parameters, delay and PDR. Furthermore, it has been found that decreasing the fragment size for low priority traffic results in reducing the delay for high priority traffic.

## 1. Introduction

Various applications of Wireless Sensor Network (WSN) in the IoT environment generate heterogeneous data which require differentiated service in terms of bandwidth, latency and reliability. WSNs comprise low power IoT devices which provide the essential monitoring functionality to various emerging applications [1,2,3,4]. Examples of such applications include healthcare [5], industrial automation and control [3], smart homes [1], smart cities [6], tracking victims and disaster management [7]. In most of these scenarios, the periodic monitoring data could be of low priority and can deal with jitter and long delay; however, there also exists high priority data such as alarms, events or commands [8], which need to be transmitted immediately. Therefore, the non-delay-tolerant traffic should be prioritized, due to its need for urgent channel access [9,10]. 

Typical WSNs were developed assuming that these networks would mostly be used for periodic monitoring applications and no strict time-constraints would be involved. However, the IoT applications which integrate WSNs have emerged as the game-changer. In time critical IoT applications such as healthcare [11], smart homes [1], and vehicle monitoring [12], even a delay on order of milliseconds could result in an accident or disaster. In this regard, a range of priority-based MAC protocols has been proposed by previous researchers which include EARS [13], TAP-MAC [5], WA-MAC [14], Urg-MAC [15], CoR-MAC [16], TraPy-MAC [17], Duo-MAC [18], WirArb [19], L-CSMA [20], MEM-MAC [9], PA-MAC [21] and PriorityMAC [22]. However, none of these schemes allow the high priority data to transmit in case the channel is occupied by low priority or less urgent data. 

In case a priority scheme or a WSN to deal with heterogeneous traffic is not implemented efficiently, the high priority real time data may starve for access due to the continuous transmission of long low priority packets. This implies the importance of pre-emptive priority, which refers to interrupting ongoing transmissions for ensuring quick channel access for high priority traffic. It is to be noted that most of the previous authors such as [13,16,23] have claimed to assign preemptive priority to the high priority traffic only by providing them a chance of transmission by holding the lower priority packets in the queue; however, no mechanism has been developed to interrupt the ongoing transmission of lower priority traffic. Clearly, in the existing protocols, the high priority traffic must wait for the channel to become free before it could begin transmission.

In this paper, we propose a simple yet novel fragmentation scheme (FROG-MAC) for transmission of heterogeneous data in WSNs; we define heterogeneous traffic as traffic which has different arrival rates. Furthermore, we assign different priorities to each traffic type. It is proposed to transmit the low priority data by splitting the packets into multiple fragments. The medium would be idle between the transmission of fragments for a certain duration (termed as ‘interruptible period’). This interruptible period could be used by the high priority traffic to begin its transmission. In this work, we present the high-level design of MAC protocol FROG-MAC and validate the performance improvement brought by the suggested scheme using MATLAB simulations.

The major contributions of this work are as follows:

(1) We propose a high-level design of fragmentation scheme (FROG-MAC) for heterogeneous traffic in WSNs which allows interruption of ongoing transmissions by high priority traffic.
(2) We develop a high-level MATLAB implementation of the proposed scheme and validate the advantages in terms of delay and PDR for the high priority traffic.

The rest of this paper has been organized as follows: Section 2 presents a brief literature review, Section 3 describes the proposed design of FROG-MAC; Section 4 presents the high-level implementation details for FROG-MAC; Section 5 describes the experimental settings; Section 6 presents the findings and analysis and finally, Section 7 concludes the work and offers directions for future research.

## 2. Relevant Work

Due to the ever-increasing pace of IoT and WSN deployment in the real-world, many authors have proposed prioritization schemes for dealing with heterogeneous traffic. Some of the recent schemes are reviewed in this section. 

Traffic Priority-based Channel Access Technique (TP-CAT) is proposed for improving the delay for critical data in healthcare scenarios [24]. The scheme is developed over 802.15.6 and aims at reducing the transmission delay as well as user conflicts for the critical data. Separate algorithms for dealing with data of low and high thresholds have been developed. Furthermore, the protocols are made efficient through introducing a novel back-off scheme named as Random Overlapping Back-off value Avoidance (ROBA) which ensures selection of random value by the nodes during back-off process. Although the protocol could efficiently reduce access delay for the critical data, the interruption of ongoing transmission is not considered. 

Data Prioritization and Capacity Assignment (DPCA) analyses the capacity of each routing path and ensures delivering high priority data in the presence of congestion [25]. The novel contribution of the scheme is to analyze the capacity of routing path and network in conjunction with the priority of data. In case the network is congested, the data of higher priority is ensured to be transmitted to the sink, whereas that of lower priority is discarded. On the other hand, efficient resource usage is also guaranteed as in case the network is not totally consumed, the bandwidth could be assigned to the lower priority data instead of waiting for the high priority data. The drawbacks of this work include that the testbed implementation or simulation of the scheme is not provided, and preemption of ongoing transmission is not discussed. 

Traffic-Adaptive Priority-based MAC (TAP-MAC) proposed an adaptive super-frame structure in order to provide the nodes with regular and emergency data with optimal channel access [5]. The channel access period (CAP) has been divided into two categories, where CAP1 is reserved for the emergency and CAP2 can be accessed by both the emergency and normal traffic. TAP-MAC hence improves the chances for channel access for the emergency traffic as it would have shorter contention window values, however, since the CAP is partially shared with low priority traffic, there is a certain probability that the nodes with low priority data would win the channel even in the presence of emergency traffic. Moreover, no mechanism has been suggested to interrupt the ongoing transmission of normal traffic. 

Urg-MAC has been specifically developed for dealing with the urgency challenges in Wireless Multimedia Sensor Networks [15]. Various mechanisms for offering priority to the urgent traffic have been implemented at the application and link layers. The traffic is divided and subdivided into different categories of real-time and non-real-time and the mechanisms of traffic rate adjustment, contention window adaptation, duty-cycle reduction and multimedia message passing have been applied. Although the protocol integrated some of the famous prioritization schemes for offering priority to the urgent traffic, the interruption of ongoing transmission is still not possible. 

A priority guaranteed MAC protocol has been developed in [26] specifically for improving the network lifetime for Wireless Body Area Networks (WBAN). The protocol categorizes the traffic into different priorities and uses the modified frame structure of 802.15.4 to efficiently manage the transmission of prioritized traffic. Furthermore, the novel component of wakeup radio has also been used in this work. Although the protocol achieves energy efficiency and reduced delay for the prioritized traffic, no mechanism to interrupt the ongoing transmission on radio has been proposed. Also, the protocol design relies on the wakeup radio which is not embedded with majority of the sensor motes today.

Priority-based Fairness Rate Control (PFRC) adjusts data rates for non-real time traffic based on the network congestion [27]. The priority of traffic as well as the fair assignment of bandwidth are used for allocating resources. Tree topology has been used and priority has been assigned to the nodes based on their level in the hierarchy. Hence, the three units of Differential Rate Adjustment Unit (DRAU), Congestion Detection Unit (CDU), Priority Adjustment Unit (PAU) and Fairness Control Unit (FCU) provide input for rate control decision making. The major limitations of the protocol lie in its priority assignment based on the node level rather than the traffic type in the node. Furthermore, it is also not possible for the nodes in PFRC to interrupt the ongoing transmission. 

Priority-based Adaptive MAC (PA-MAC) is developed for (WBAN) in order to customize the IEEE 802.15.4 for medical applications [21]. PA-MAC dynamically allocates slots and contention window to the traffic based on its priority level. Different categories of traffic such as emergency, on-demand, normal, non-medical have been assigned priority based on their delay tolerance level. The major performance gap in PA-MAC is the lack of interruption opportunity for the emergency traffic and the sharing of transmission slots between the high & low priority data.

Priority-Based Data Gathering Framework for WSN using Unmanned Aerial Vehicles (UAV) has been developed in [28], with the aim of improving the data gathering method. The nodes have been assigned different priorities based on their locations within the UAV coverage area. The priorities have been assigned to nodes, using different contention window values. The authors have also proposed a supporting routing protocol to optimize the energy performance of network. The interruption for ongoing traffic is also not included in this approach. 

Interrupt-MAC (I-MAC) has been developed for WBANs by inserting short interruption slots in the super frame which could reduce the data delivery delay for urgent medical traffic [29]. Most of the time slots are allocated to the nodes which have regular periodic data, however, during the short interruption slot, the coordinator can break the executing super frame to start the new one with real-time requirement. Although I-MAC provides a higher chance for emergency traffic to access the channel as compared to conventional super frame-based schemes, the nodes still need to wait for the coordinator to interrupt the ongoing transmission and they could not send data on their own.

Game theory-based approach has been used to assign priorities to the nodes in WBAN in order to efficiently deal with critical data [30]. The gap identified by the authors is that conventional protocols in WBAN deal similarly with the packets with regular health monitoring data and packets with critical data, which results in high average time for the data with critical information. Hence, the authors proposed Priority-based Allocation of Time Slots (PATS) which assigns priority to the nodes based on the criticality of information. Hawk-dove game model has been used for priority assignments. The proposed scheme may guarantee low average delay for the critical health monitoring data; however, the complexity of the scheme is relatively higher as compared to other proposed schemes. The miniature WSN nodes can only handle limited complexity due to the limit on computing resources. Furthermore, no support for interrupting the ongoing low priority transmission is offered. 

RushNet focuses on efficient delivery of heterogeneous data in the saturated WSN [8], where the rate of data generation is assumed to be close to the available network bandwidth. The high priority data has been assumed to be sporadic but urgent, whereas the authors classified low priority traffic with high volume but to be delay tolerant. The protocol does not need co-ordination of schedules and is designed to perform well for the large scale WSN. The two schemes of bulk transfer and capture effects have been used. RushNet first develops a network backbone on which high volume of low priority data is transmitted and high priority packets are then sent without the need to stop ongoing transmissions; capture effect is used for this purpose which is the property of a radio to be able to decode a stronger signal from one transmitter, even if other weaker signals are also available in the range. Thus, the radio capture effect enables the nodes in RushNet to send the low volume high priority data anytime. Although RushNet allows higher priority data to be transmitted even if the channel is busy, two separate transceivers are required by the protocol, which is its major drawback. 

The major research gap identified in the light of the above literature analysis is the lack of a MAC protocol which could offer a true preemptive priority mechanism for heterogeneous traffic in WSNs. Table 1 summarizes the pros and cons of the priority schemes reviewed in this paper.

## 3. Proposed Design

FROG-MAC is specifically designed to facilitate the WSN and IoT applications based on heterogeneous traffic. As previously described, for the situations where the emergency traffic could not be delivered in time, the major purpose of WSN deployment could be negatively affected and the required operation could either never take place or be delayed. Another issue found in the previous protocols was that they kept the channel reserved for high priority traffic which could result in resource wastage. The proposed protocol FROG-MAC sends the low priority data in fragments with pauses in between in order to facilitate the emergency traffic. In case some other nodes within the range generate high priority data, they could immediately transmit benefiting from the pauses between the fragments of low priority data. In comparison, the high priority data is not fragmented, and the complete packet is sent at once. Therefore, FROG-MAC would offer the opportunity to emergency traffic for immediate delivery even if the low priority traffic was transmitting. In addition, FROG-MAG resolves the issue of resource wastage as well by scheduling the transmission of low priority traffic when the channel is available, instead of keeping the channel reserved for high priority data. 

**Figure 1:**
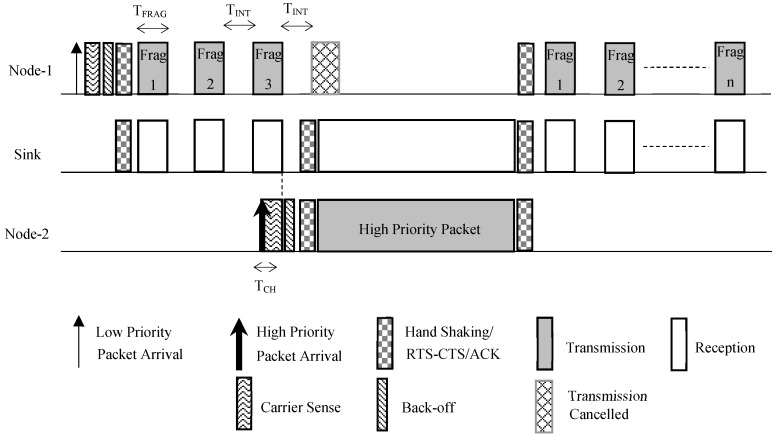
FROG-MAC Design.

For illustration of the FROG-MAC design, two types of traffic have been assumed, where one depicted regular traffic (low priority) and the other has been used for demonstrating the emergency traffic (high priority). For illustrating the low priority or regular traffic, traffic of Type *T*_1_ with Priority *P*_1_ has been assumed, which is modeled as CBR traffic with high data rate. In contrast, for the high priority, type *T*_2_ with Priority *P*_2_ has been used which has Poisson arrivals and a lower data rate. These traffic distributions have been assumed due to the fact that emergency packets are less likely to be generated, and also whenever they would be generated, they would not have a regular pattern; similar assumptions have been found in the previous work [8]. The design of FROG-MAC has been illustrated by Figure 1, where it has been assumed that node 1 transmits low priority (*T*_1_) traffic, whereas node 2 is shown to transmit high priority (*T*_2_) traffic to a common sink (node S). Furthermore, it has been assumed that communication has been established between the transmitted nodes and sink using some handshaking mechanism; RTS/CTS (handshaking) mechanism is out of scope for this discussion.

As shown in Figure 1, node 1 begins the transmission process by performing carrier sensing for a checking period *T_CH_*. Upon finding the channel idle, it performs back off and then begins transmission of its low priority packet in fragments after establishing the communication using some handshaking scheme. The size of each fragment is S_FRAG_ (bytes) and duration of each fragment is *T_FRAG_*. Between two consecutive fragments, an interval ‘interruptible period’ *T_INT_* is assumed in order to enable the node with high priority traffic to access the medium. In case no high priority data is generated between this period, all the pending fragments of low priority would transmit. When high priority data is generated at some node, it performs carrier sense for ‘checking period’ *T_CH_* (*T_CH_* < *T_INT_*); if the channel is found to be idle, it performs back-off and transmit. On the other hand, if a node with high priority data does not find the channel idle during *T_CH_*, it continues to listen to the channel and transmits as soon as the channel becomes available (this case has been illustrated in Figure 1). It is to be noted that high priority data is not fragmented and is sent as a single unit. At present, low and high priority data are not separately stored; hence, all the fragments of low priority data which had been transmitted prior to the channel interruption by high priority data are retransmitted once the channel becomes available. 

In the scenario shown in Figure 1, the case is depicted where node 2 generates high priority packet during the transmission of fragments by node 1. Node 2 begins listening to the channel; when it detects the channel idle for the duration *T_CH_*, it begins sending its packet. The FROG-MAC is a pseudo-preemptive protocol because although it reduces the waiting time for high priority data significantly as compared to the traditional preemptive protocols, this data still has to wait for the transmission of short fragment which is already in transmission.

Once the high priority transmission gets completed, the low priority data continues its transmission. The future components of FROG-MAC would include acknowledgement and buffer mechanisms. The buffer would help to store the previously received low priority fragments, so their retransmission could be avoided.

Figure 1 illustrated the case where node 2 had high priority data. In case if node 2 had low priority data, it would not transmit because during the handshaking, it would already know that the medium would be occupied due to the data transmission by node 1. When the node 1 finished its transmission and receives acknowledgement (also received by node 2), both nodes would run back-off process in their attempt to win the channel. Similarly, in case both the nodes have high priority data at same instant, random value of back-off would decide the winning node. 

It is to be noted that the major focus of FROG-MAC is to reduce the channel access delay for the high priority traffic in the time-critical applications of WSN. The delay is expected to reduce by the proposed fragmentation scheme. However, the drawbacks of FROG-MAC include high energy cost and the single hop topology. The impact of these limitations would be minimized by authorizing the high priority traffic to immediately access the channel. Hence, the scope of FROG-MAC might be limited to the application scenarios where energy is not a constraint and reducing delay is a crucial requirement. 

## 4. Implementation Details

Since the focus of this paper is on presenting a proof of concept for the design of FROG-MAC, the high-level implementation of the protocol has been developed in MATLAB. It is to be noted that the packets are not actually transmitted or received but the delay and PDR is calculated based on the values stored in arrays. Furthermore, other schemes have also been implemented in order to validate the performance of FROG-MAC. This section presents some details of the algorithms for the interest of reader. 

### 4.1. Generating Heterogeneous Arrivals

The arrival patterns for CBR and Poisson arrivals (with exponentially distributed inter-arrivals) have been generated using the Algorithm 1. The values of instants at which arrival occurs are stored in arrays and then subsequently used for calculations of delay and PDR.

Following MAC schemes at high level have been studied and implemented in this paper:

(a) First Come First Serve (FCFS)

The delay analysis has been performed when no priority was assigned to any of the traffic types. The packets which are generated first, transmit first regardless of their type. This is the simplest case and was implemented to validate the performance improvement brought by FROG-MAG. Implementation of this scheme along with the delay calculation for each traffic type has been described in Algorithm 2. 

(b) Prioritization without Fragmentation

In this scheme, the packets have been prioritized but fragmentation of low priority packets is not implemented. The high priority packets are transmitted first, even when there are low priority packets present in queue. However, the high priority traffic cannot interrupt the ongoing transmission of low priority packets. This scenario illustrates the concept of assigning preemptive priority as defined by the previous studies, and for the sake of implementation, the fragment size for low priority is kept as 1 (so that full packets is transmitted). Implementation of this scheme is similar to the one depicted in Algorithm 3 for the case when fragment size becomes equal to packet size (which would imply that complete packet is sent as a single fragment/no fragmentation applied).

(c) FROG-MAC (Prioritization using Fragmentation)

Finally, the preliminary version of the fragmentation-based protocol FROG-MAC has been implemented, where the low priority packets have been fragmented; the high priority traffic can thus interrupt and access the medium to begin its transmission. It has been assumed that the instants at which regular and emergency packets are generated by each node are known by every node in the network. The implementation of fragmentation scheme for low priority data has been described in Algorithm 3.


**Algorithm 1. Generating Regular and Emergency Traffic Arrivals.**

Initialize variables:Total number of ***Nodes*** = 100; ***CBR_msg_interval*** = 60 s; ***EXP_msg_interval*** = 120 s; ***Sim_Dur*** = 6000 s.Calculate number of CBR events in one node for entire Simulation duration***CBR events*** = ***Sim_Dur/CBR_msg_interval***Calculate number of EXP events in one node for entire Simulation duration***EXP events*** = ***Sim_Dur/EXP_msg_interval***Initialize variable ***node*** = 1 and a 2D-array ***CBR arrivals***Initialize ***i*** = 1.If ***i*** is greater than ***CBR events*** goto step 4-c.***CBR arrivals*** (node, i) = ***CBR_msg_interval*** * i.Increment ***i***.Goto step 4-b.Increment ***node***.If ***node*** is greater than total number of Nodes goto step 5, else goto step 4-a.Initialize ***node*** = 1 and a 2D-array ***EXP arrivals***Generate random exponential series ***exp_rand***: (mean = ***EXP_msg_interval***) of ***EXP events***.Initialize ***i*** = 1.If ***i*** is greater than ***EXP events*** goto step 5-d.***EXP arrivals*** (node, i) = ***EXP arrivals*** (node, i-1) + ***exp_rand*** (*i*).Increment ***i***.Goto step 5-c.Increment ***node***.If ***node*** is greater than total number of Nodes goto step 6, else goto step 5-a.End



**Algorithm 2. FCFS and Corresponding Delay Calculation.**
1. Sort the 2D-array ***CBR arrivals*** in ascending order and convert it into a 1-D array ***CBR arrivals***2. Set ***Total_CBR_events*** = elements in ***CBR arrivals.***3. Sort the 2D-array ***EXP arrivals*** in ascending order and convert it into a 1-D array ***EXP arrivals***4. Set ***Total_EXP_events*** = elements in ***EXP arrivals.***5. Set ***Delay*** = propagation time + packetization time6. Initialize variables ***Pointer***, ***temp*** = 0; ***i*** = 0; ***j*** = 0. a. if ***i*th *CBR arrival*** occurs before ***j*th *EXP arrival***   • set ***Pointer*** = ***i*th *CBR arrival*** b. else   • set ***Pointer*** = ***j*th *EXP arrival***7. if ***i*** < ***Total_CBR_events*** and ***j*** < ***Total_EXP_events*** a. if ***i*th *CBR arrival*** occurs before ***j*th *EXP arrival***   • if ***Pointer*** > ***CBR arrivals*** (***i***)    ➢ ***temp*** = difference between ***Pointer*** and ***i*th *CBR arrival***    ➢ ***Pointer*** = ***Pointer*** + ***Delay***   • else    ➢ ***Pointer*** = ***i*th *CBR arrival***   • Calculate ***CBR delay*** (***i***) = ***Delay*** + ***temp***   • Increment ***i*** b. if ***j*th *EXP arrival*** comes before ***i*th *CBR arrival***   • if Pointer > ***EXP arrivals*** (***j***)    ➢ ***temp*** = difference between ***Pointer*** and ***j*th *EXP arrival***    ➢ ***Pointer*** = ***Pointer*** + ***Delay***   • else    ➢ ***Pointer*** = ***EXP arrivals*** (***j***) + ***Delay***   • Calculate ***EXP delay*** (***j***) = ***Delay*** + ***temp***   • Increment ***j*** c. ***temp*** = 0; Goto step 7.8. if ***i*** > ***Total_CBR_events*** and ***j*** < ***Total_EXP_events*** a. if ***Pointer*** > ***EXP arrivals*** (***j***)   • ***temp*** = difference between ***Pointer*** and ***j*th *EXP arrival***   • ***Pointer*** = ***Pointer*** + ***Delay*** b. else   • ***Pointer*** = ***EXP arrivals*** (***j***) + ***Delay*** c.  Calculate ***EXP delay*** (***j***) = ***Delay*** + ***temp*** d. Increment ***j***; ***temp*** = 0; Goto step 8.9. if ***j*** > ***Total_EXP_events*** and ***i*** < ***Total_CBR_events*** a. if ***Pointer*** > ***CBR arrivals*** (***i***)   • ***temp*** = difference between ***Pointer*** and ***i*th *CBR arrival***   • ***Pointer*** = ***Pointer*** + ***Delay*** b. else   • ***Pointer*** = ***CBR arrivals*** (***i***) + ***Delay*** c. Calculate ***CBR delay*** (***i***) = ***Delay*** + ***temp*** d. Increment ***i***; ***temp*** =0. e. Goto step 910. Calculate ***max_CBR_delay*** using array ***CBR delay***.11. Calculate ***max_EXP_delay*** using array ***EXP delay***.12. End.


**Algorithm 3. Delay Calculation for Prioritization with Fragmentation Scheme.**
1. Sort the 2D-array ***CBR arrivals*** in ascending order and convert it into a 1-D array ***CBR arrivals***2. Set ***Total_CBR_events*** = elements in ***CBR arrivals.***3. Sort the 2D-array ***EXP arrivals*** in ascending order and convert it into a 1-D array ***EXP arrivals***4. Set ***Total_EXP_events*** = elements in ***EXP arrivals.***5. ***Total Frags*** = Packet Size / Fragment Size6. Set ***Delay*** = propagation time of one Fragment + packetization time of one Fragment7. Set ***DelayPkt*** = propagation time of a Packet + packetization time of a Packet8. ***FragPtr*** = 19. ***Initialize*** variables ***Pointer***, ***temp*** =0; ***i*** = 0; ***j*** = 0. a. if ***i*th *CBR arrival*** occurs before ***j*th *EXP arrival***   • set ***Pointer*** = ***i*th *CBR arrival*** b. else   • set ***Pointer*** = ***j*th *EXP arrival***10. if ***i*** < ***Total_CBR_events*** and ***j*** < ***Total_EXP_events*** a. if ***i*th *CBR arrival*** occurs before ***j*th *EXP arrival***  i. if ***Pointer*** > ***CBR arrivals*** (***i***)   ➢ ***temp*** = difference between ***Pointer*** and ***i*th *CBR arrival***   ➢ ***Pointer*** = ***Pointer*** + ***Delay***  ii. else   ➢ ***Pointer*** = ***i*th *CBR arrival*** + ***Delay***  iii. Calculate ***CBR delay*** (***i***) = ***Delay*** + ***temp***  iv. Increment ***FragPtr***  v. if ***FragPtr*** < ***Total Frags***   ➢ if ***Pointer*** < ***EXP arrivals*** (***j***)    a. ***Pointer*** = ***Pointer*** + ***Delay***    b. Calculate ***CBR delay*** (***i***) = ***CBR delay*** (***i***) + ***Delay***    c. Goto 10-a-iv.   ➢ else    a. ***temp*** = difference between ***Pointer*** and ***j*th *EXP arrival***    b. ***Pointer*** = ***Pointer*** + ***DelayPkt***    c. Calculate ***EXP delay*** (***j***) = ***DelayPkt***+ ***temp***    d. Increment ***j***  vi. else   ➢ Increment ***i***  vii. ***temp*** = 0  viii. Goto step 10
 b. if ***j*th *EXP arrival*** occurs before ***i*th *CBR arrival***  i. if ***Pointer*** > ***EXP arrivals*** (***j***)   ➢ ***temp*** = difference between ***Pointer*** and ***j*th *EXP arrival***   ➢ ***Pointer*** = ***Pointer*** + ***DelayPkt***  ii. else   ➢ ***Pointer*** = ***EXP arrivals*** (***j***) + ***DelayPkt***  iii. Calculate ***EXP delay*** (***j***) = ***DelayPkt*** + ***temp***  iv. Increment ***j*** c. ***temp*** = 0. d. Goto step 10.11. if ***i*** > ***Total_CBR_events*** and ***j*** < ***Total_EXP_events*** a. if ***Pointer*** > ***EXP arrivals*** (***j***)  i. ***temp*** = difference between ***Pointer*** and ***j*th *EXP arrival***  ii. ***Pointer*** = ***Pointer*** + ***DelayPkt*** b. else   • ***Pointer*** = ***EXP arrivals*** (***j***) + ***DelayPkt*** c. Calculate ***EXP delay*** (***j***) = ***DelayPkt*** + ***temp*** d. Increment ***j***; ***temp*** =0. e. Goto step 11.12. if ***j*** > ***Total_EXP_events*** and ***i*** < ***Total_CBR_events*** a. if ***Pointer*** > ***CBR arrivals*** (***i***)  i. ***temp*** = difference between ***Pointer*** and ***i*th *CBR arrival***  ii. ***Pointer*** = ***Pointer*** + (Delay * Total Frags) b. else   • ***Pointer*** = ***CBR arrivals*** (***i***) + (Delay * Total Frags) c. Calculate ***CBR delay*** (***i***) = (Delay * Total Frags) + ***temp*** d. Increment ***i***; ***temp*** =0. e. Goto step 1213. Calculate ***max_CBR_delay*** using array ***CBR delay***.14. Calculate ***max_EXP_delay*** using array ***EXP delay***.15. End.

## 5. Experimental Settings

Due to the nature of the emergency traffic, we have selected performance criteria of ‘maximum delay’ for performance evaluation instead of average delay. This choice has been made because in case of the critical applications such as healthcare, the user should be aware of the maximum delay which could be incurred by the protocol. In addition, we have also studied the influence of varying packet size on the Packet Drop Ratio (PDR) for traffic of both types. For illustrating the PDR, we have considered the packets which faced a delay of 2 s or more as lost. However, these packets have still been incorporated for the delay calculations and therefore, we find much higher delay values in the experimental results. 

**Figure 2:**
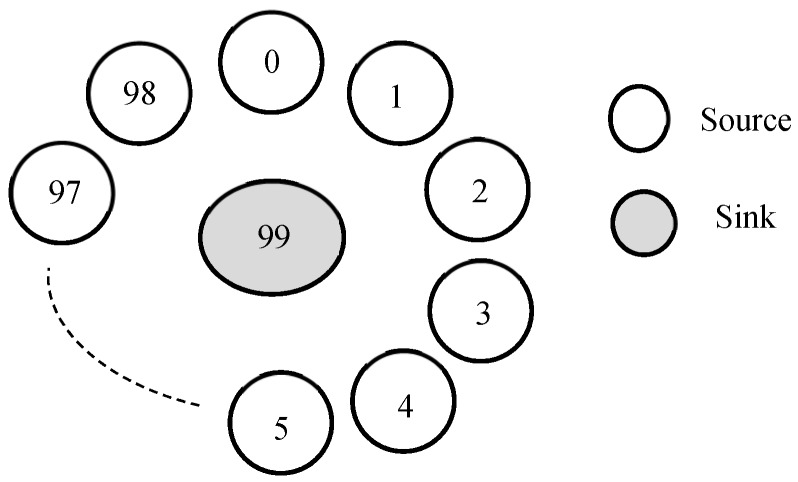
Topology Settings.

Experimental set up of 100 nodes, arranged in single hop star topology was used, as shown in Figure 2. Similar topologies have been used by previous researchers for conducting performance analysis of MAC schemes dealing with heterogeneous traffic [9,17,31]. The topology used fundamentally depicts the communication between the cluster head and one-hop distant nodes. 

As shown in Figure 2, node 99 was the sink, whereas multi-sender environment was considered with nodes 0 to 98 generating heterogeneous traffic of two types at specified intervals. Traffic of type-1 (*T*_1_) was generated at Constant-Bit Rate (CBR) to represent the periodic monitoring applications of IoT. On the other hand, Type-2 (*T*_2_) traffic was generated at exponentially distributed inter arrival periods (Poisson arrivals) to depict the emergency traffic. The traffic modeling has been done based on assumptions made in [17].

The packet size of both *T*_1_ & *T*_2_ traffic was assumed to be same, whereas the average arrival rate of *T*_2_ has been considered lower as compared to that of *T*_1_. The parameters of packet size and transmission time have been assumed based on existing implementations of SCP-MAC [32] & ADP-MAC [33]. Also, the different packet arrival rates of two traffic types have been selected based on the assumptions made in previous literature [22,23]. Table 2 summarizes the experimental settings.

## 6. Results and Discussion

This section presents the analysis of delay and PDR results for all the cases studied, i.e., First Come First Serve, Prioritization without Fragmentation and finally, Prioritization using Fragmentation. 

### 6.1. First Come First Serve (FCFS)

To begin the experimental study, the delay for both traffic types was investigated without assigning priorities. In this scheme, as soon as a packet is generated, it is transmitted subject to availability of the channel, regardless of the packet type. The delay has been computed by summing up packetization and queuing delays. Packetization delay refers to the time taken by a packet to be transmitted once it gets access to the medium. On the other hand, the queuing delay refers to the delay faced by the packet within the node while waiting for the channel; a simplified global queue has been assumed to monitor the status of packets present in each node. Hence, if there is an earlier generated packet ready for transmission at node 1, the packet which is generated later at node 2 would have to wait until the previous packet of node 1 gets transmitted.

**Figure 3:**
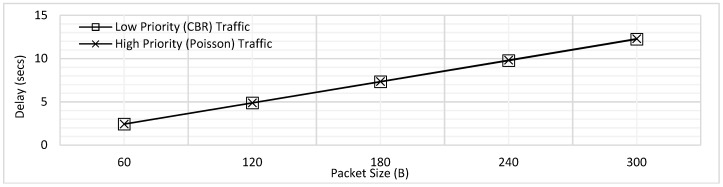
Maximum Delay of Low and High Priority Traffic for the FCFS Scheme.

Using the experimental settings shown in Table 2, the results shown in Figure 3 were obtained. Packet size was varied from 60B to 300B with increment of 60B and the maximum delay faced by CBR traffic *T*_1_, and Poisson traffic *T*_2_ has been shown. In the figure, the trend of delay is seen to be increasing with the increasing packet size. As the packet size increases, the packetization delay increases; at the same time, the queuing delay also increases as the packets must wait longer for the ongoing transmissions to get complete. The overlap for delay of both traffic types is clearly visible in Figure 3. Hence, when no priority is implemented, no difference in delay is seen for the emergency and normal traffic.

**Figure 4:**
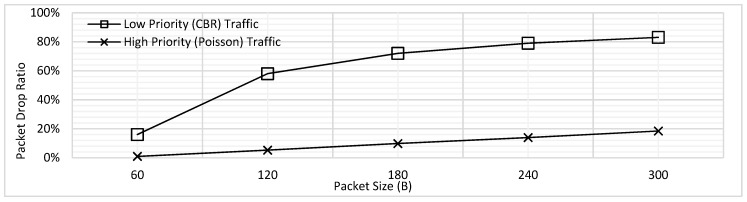
Packet Drop Ratio (PDR) of Low and High Priority Traffic for FCFS Scheme.

In addition, the PDR was also analyzed for the varying packet lengths. As illustrated by Figure 4, the PDR increases for both traffic types with the increasing packet size. The rationale behind this trend is that in case a longer packet is being transmitted, the packets in the queue must wait for long which results in increased packet drop. As mentioned above, we have assumed for all PDR results illustrated in this paper that the packets which face delay of 2 s or more are dropped. Hence, when the longer packets would be transmitting, there would be more packets which would need to wait for more than 2 s and hence the PDR increase. Secondly, Figure 4 also reveals the difference of PDR for the low and high priority traffic; the trend shows steady increase for the high priority, where the values of PDR is far higher for the CBR. The reason behind this difference in behavior is the present design where it has been assumed that all the CBR packets are generated periodically at the same time; clearly, this would result in higher delay and hence the PDR. In contrast, for the high priority traffic, the packets can be generated at each node at any point in the time, which reduces the waiting time and hence a steady increase for the values of PDR has been observed. 

### 6.2. Prioritization without Fragmentation

In this experiment, priorities were assigned to both traffic types. Low Priority *P*_1_ was assigned to Type-1 (*T*_1_) CBR traffic and High Priority *P*_2_ was assigned to Type-2 (*T*_2_) Poisson traffic. No fragmentation has been applied for the low priority traffic, and all packets are transmitted as a single unit/fragment. In case a packet of *P*_2_ is generated in any node, it is transmitted before the low priority traffic present with all the other nodes (as previously mentioned, global queue is implemented). However, the high priority traffic cannot interrupt the ongoing transmission of low priority traffic and thus, it has to wait until the low priority packet fully gets transmitted. This case represents the prioritization mechanisms developed by majority of previous researchers.

**Figure 5:**
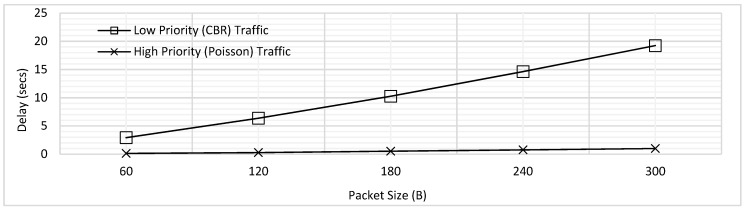
Comparison of Delay for Prioritization without Fragmentation.

The delay comparison for traffic of both priorities for this experiment has been illustrated in Figure 5. The delay for both traffic types is found to be increasing with increasing packet size due to the increased packetization and queuing delays. The maximum delay for low priority traffic is found to be considerably higher than high priority traffic, as per the expectation. The low priority packets wait for all the high priority traffic to transmit first, which is the cause behind the higher delay. In contrast, as the high priority packets are lower in number and also generate at random intervals, they face very less delay. 

**Figure 6:**
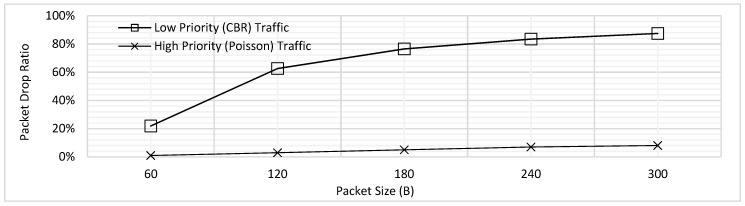
Comparison of PDR for Prioritization without Fragmentation.

Figure 6 depicts the PDR trends for low and high priority traffic for the prioritization without fragmentation scheme. As described earlier in Section 4.1, the PDR for high priority traffic remain low as compared to the CBR due to the packet generation rate and associated high probability of finding channel engaged. 

**Figure 7:**
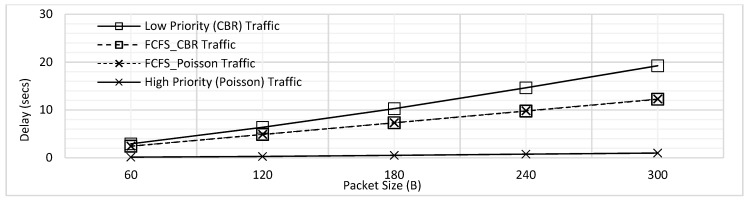
Delay Comparison for with and without Prioritization (Figure 3 and Figure 5 Combined).

**Figure 8:**
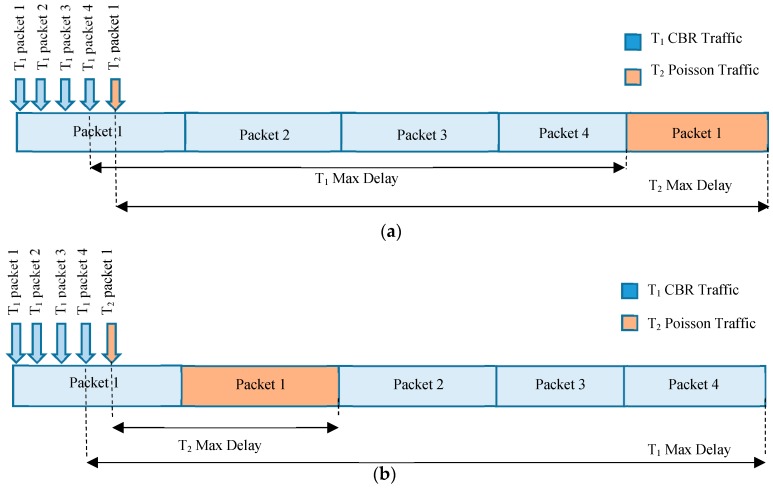
Delays for Low (CBR) and High (Poisson) Priority Traffic. (**a**) FCFS (**b**) Prioritization.

Figure 7 combines the results presented in Figure 3 and Figure 5 to illustrate the effect of prioritization. It has come to light that the maximum delay for *T*_1_ traffic has slightly increased whereas there is more significant decrease in the delay of *T*_2_ traffic. This trend can be explained further by considering an example case where 4 packets of *T*_1_ and one packet of *T*_2_ are to be transmitted. Let us assume that first packet of *T*_1_ was already transmitting and there were three more packets of *T*_1_ in the queue at the instant when a packet of *T*_2_ is generated, as shown in Figure 8.

For the case of FCFS illustrated in Figure 6a, the packet of *T*_2_ has to wait for all the packets of *T*_1_ which were generated earlier resulting in a high maximum delay; when the prioritization is applied, *T*_2_ packet would just need to wait for the completion of ongoing transmission of the first packet of *T*_1_, which results in a significant reduction of maximum delay. On the other hand, the case is opposite for the traffic of *T*_1_. For FCFS, all the packets were served in the sequence they were generated in, whereas for the prioritization illustrated by Figure 6b, the packet would have to wait for transmission of one packet of *T*_2_. Hence, the delay advantage for the *T*_2_ traffic is high with only a little cost for *T*_1_ traffic.

**Figure 9:**
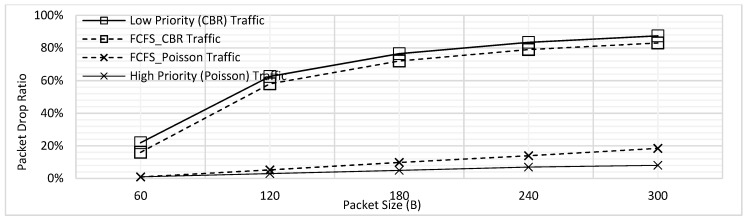
PDR Comparison for with and without Prioritization (Figure 4 and Figure 6 Combined).

Similarly, Figure 9 combined the results earlier presented in Figure 4 and Figure 6. As seen from the figure, the PDR for CBR traffic is not significantly increased, however, that of Poisson traffic is further reduced. Hence, it has been found in this section that prioritization without fragmentation provides better performance for high priority traffic when compares to FCFS. 

### 6.3. Prioritization using Fragmentation

Fragmentation of low priority traffic has been implemented in this experiment. Initially, the fragment size was kept as 1 B for each packet size (for example, 60 fragments would be created for a single data packet when the packet size is 60 B), and subsequently, the fragment size was increased to reach the full packet size. The rationale behind selecting 1 B as the smallest fragment size is the fact that the intended testbed is mica2 platform; mica2 motes use CC1000 radio which can transmit 1 B as the smallest unit. We first discuss the experiments for fragment size = 1 B.

#### 6.3.1. Fragment Size = 1B

**Figure 10:**
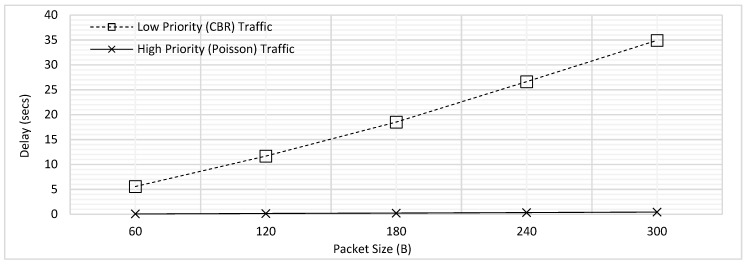
Delay for Prioritization using Fragmentation (Fragment Size = 1B).

In this experiment, after transmitting one byte of the *T*_1_ data packet, the transmission is paused for the duration of *T_INT_*, in order to facilitate the transmission of emergency/high priority traffic. In case a packet of *T*_2_ is generated at any node, it would listen to the medium for *T_CH_*, if the channel is free it immediately transmit. In case channel is busy with some ongoing transmission of low priority packet, the node continues to listen even after *T_CH_* and begins its transmission as soon as the medium gets free during *T_INT_*; this causes the transmission of *T*_1_ packet to terminate. Hence, the delay of high priority traffic is reduced as it does not need to wait for the entire packet of low priority to complete its transmission and it could instead access the channel after transmission of short fragment. The packet of *T*_1_ which was interrupted, shall be completely retransmitted once the channel is available. It is to be noted that the protocol in its present form, does not buffer the transmitted fragments at the receiver’s end; the bytes which were previously transmitted are considered lost. The influence of applying fragmentation scheme on the delay performance of low and high priority traffic for varying packet size is shown in Figure 10.

**Figure 11:**
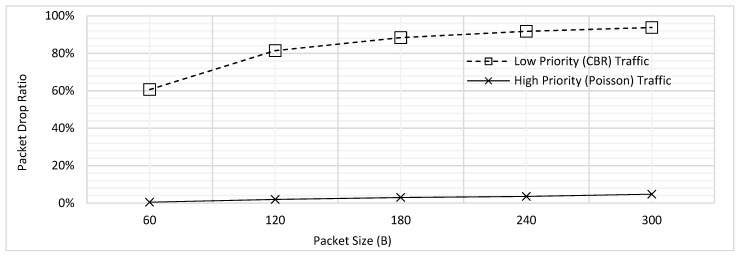
PDR for Prioritization using Fragmentation (Fragment Size = 1B).

The effect of applying fragmentation on the PDR of low and high priority traffic has been illustrated in Figure 11. As expected, the PDR is much higher for CBR with fragmentation because now the packets will have to wait even longer due to the channel being interrupted during the transmission of a single packet. 

**Figure 12:**
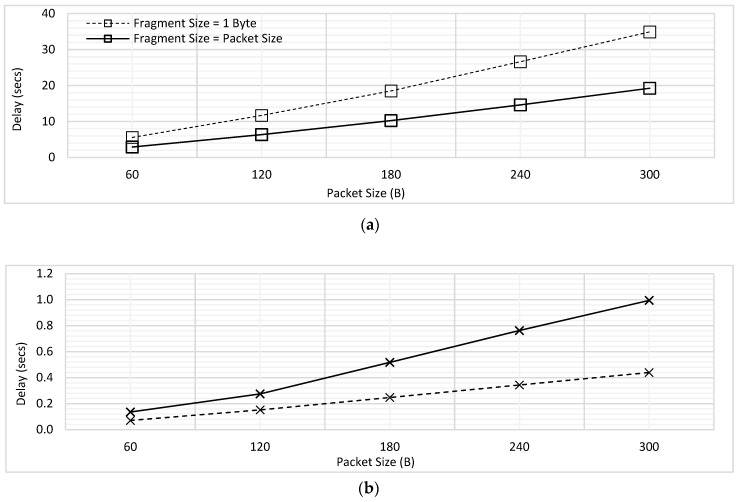
Comparing the Influence of Fragmentation on Delay (**a**) Low Priority Traffic (**b**) High Priority Traffic.

The delay performance for the low and high priority traffic with & without fragmentation has been shown in Figure 12. For the case of ‘without fragmentation’, complete packet is transmitted as a single unit; in other words, fragment size is kept equal to the packet size. On the other hand, fragment size is kept as 1B for the case of ‘with fragmentation’. Figure 12a shows that for low priority CBR traffic, the delay increases due to fragmentation, whereas Figure 12b indicates the delay advantage for high priority Poisson traffic.

**Figure 13:**
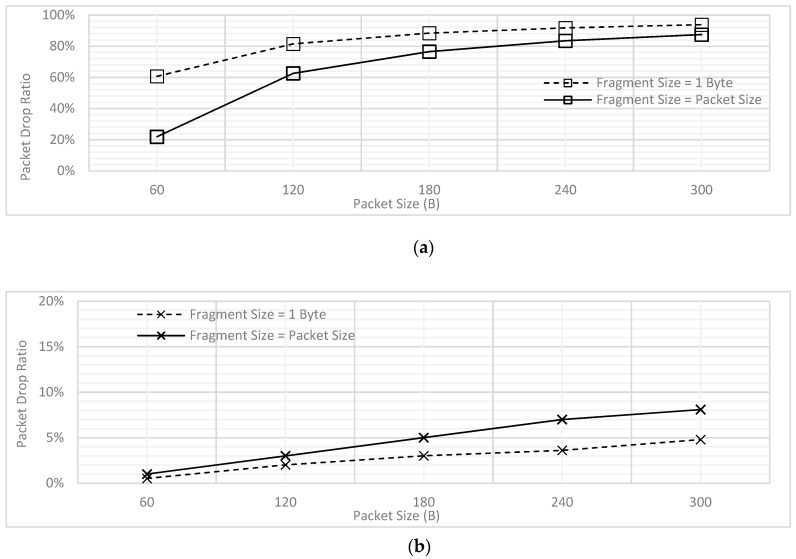
Comparing the Influence of Fragmentation on PDR (**a**) Low Priority Traffic (**b**) High Priority Traffic.

Subsequently, Figure 13 provides comparison of PDR for low and high priority traffic for the cases of with and without fragmentation. When fragment size is set to 1B, the low priority traffic faces high PDR and high priority traffic faces lower PDR. In contrast, when the fragment size reaches to full packet size, the delay trends become opposite for both the priorities because now the lower priority traffic gets higher chances of complete transmission before getting dropped. An interesting finding observed in Figure 13a is that for low priority traffic, as the packet size is increased, the delay trend converges for different fragment sizes. 

This is because even if the fragment size is increased to the full packet size, the PDR will still be high as more packets will be dropped due to waiting more than 2 s; this result confirms the findings earlier depicted in Figure 4. On the other hand, in Figure 13b, when the packet size was increased, the difference between the PDR values for with and without fragmentation scheme increased as instead of waiting for the full packet of 300B to be transmitted, the high priority traffic would now wait only for transmission of 1B fragment. 

#### 6.3.2. Influence of Varying Fragment Size

**Figure 14:**
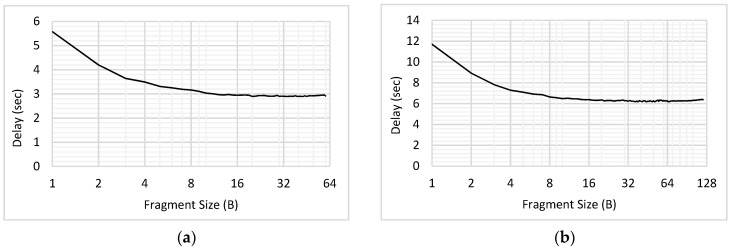

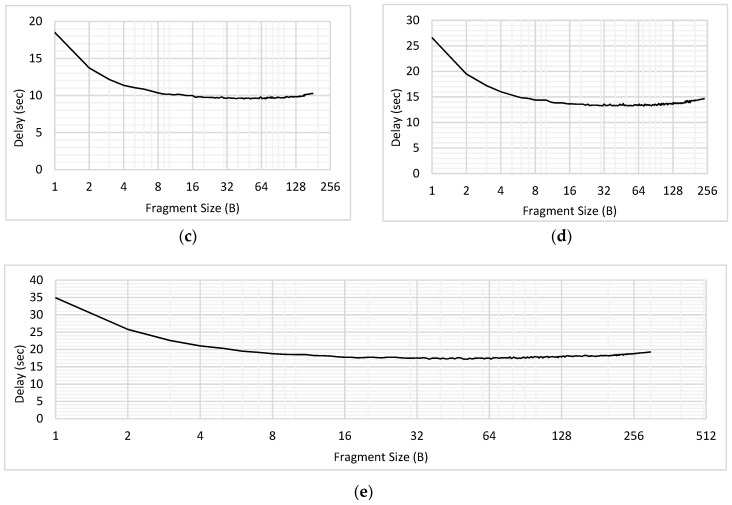
Influence of Varying Fragment Size for Low Priority (CBR) Traffic. (**a**) Packet Size = 60B; (**b**) Packet Size = 120B; (**c**) Packet Size = 180B; (**d**) Packet Size = 240B; (**e**) Packet Size = 300B.

The influence of varying fragment size S_FRAG_ for the traffic of both priorities was studied in this experiment. The results of delay for low priority CBR traffic have been shown in Figure 14. Packet size was assumed to be 60, 120, 180, 240 and 300 bytes. For each packet, the fragment size was varied from 1B to the maximum packet size (when the fragment size = 1B, maximum number of fragments will be transmitted, and when fragment size = packet size, the complete packet is transmitted as a single unit). As shown in the Figure 14, for each value of the packet size, the delay reduces with the increasing fragment size; this happens because the number of interruptible periods between the fragments reduces, which offers a lower chance to high priority traffic to take up the medium during the ongoing transmission.

In each of the graphs shown in Figure 14, the sharp decline in the value of delay has been seen initially, whereas the trend becomes steady for the higher values of fragment sizes. Let us consider the variation of fragment sizes in Figure 14a for the explanation of trend, where the packet size was 60B. Initially, S_FRAG_ was increased from 1B to 2B and then from 2B to 3B. Considering S_FRAG_ = 1B, there would be 60 fragments and hence 60 interruptible periods, whereas when S_FRAG_ increases to 2B, the number of interruptible periods reduces to 30. This shows that there occurs a difference of 30 interruptible periods only by changing S_FRAG_ by 1B. However, when the S_FRAG_ increases from 2B (with 30 interruptible periods) to S_FRAG_ = 3B (with 20 interruptible periods); only 10 interruptible periods are reduced with the increment of 1B in S_FRAG_. As S_FRAG_ keeps on increasing, the difference in number of interruptible periods further reduces. Hence, initially we see a sharp decline in delay due to having higher differences in the number of interruptible periods, which gradually reduces to exhibit approximately constant delay. 

Furthermore, a slight increase in the value of delay for *T*_1_ traffic has been observed for high S_FRAG_, in each of the Figure 14b–e. For the high value of S_FRAG_, the interruption may occur only after transmitting a large portion of *T*_1_ packet. For example, if packet of *T_2_* interrupts the transmission of *T*_1_ when S_FRAG_ = 50 in Figure 14b, the time which was spent in transmitting the 50 B of *T*_1_ data would be wasted. Due to the present limitation in the implementation, the bytes of *T*_1_ which are transmitted prior to getting interrupted are considered lost. Therefore, with the increasing S_FRAG_, the maximum delay is found to be increasing. This contradicts with the initial trends shown in Figure 14, where the delay is seen to be decreasing with increasing S_FRAG_; as S_FRAG_ increases and becomes equal to the packet size, the delay reduces as the packet is transmitted as a single unit without being interrupted. However, if the packet gets interrupted anywhere in between, it would face higher delay as compared to the case where S_FRAG_ was low.

**Figure 15:**
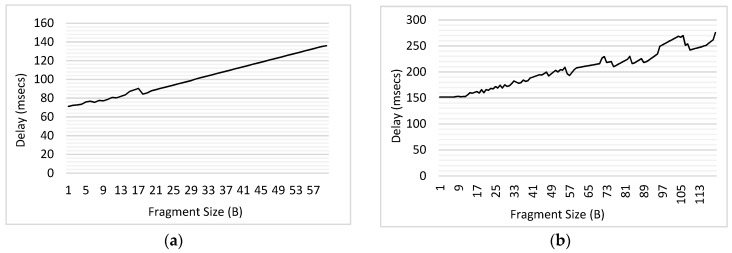

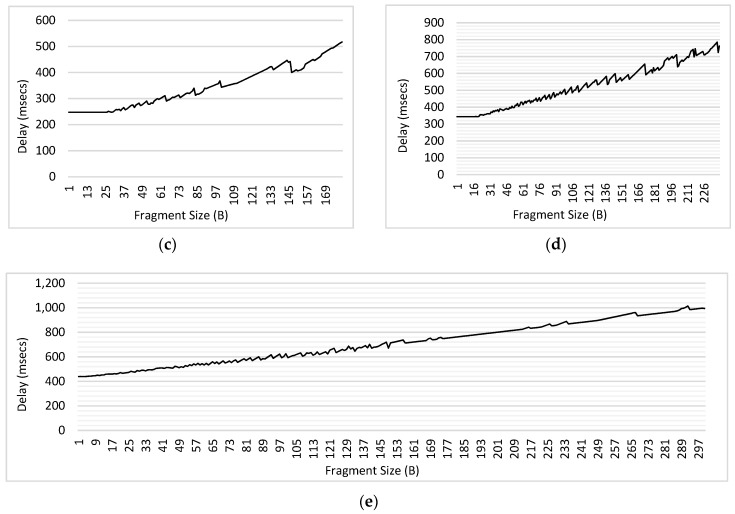
Influence of Varying Fragment Size for High Priority (Poisson) Traffic. (**a**) Packet Size = 60B; (**b**) Packet Size = 120B; (**c**) Packet Size = 180B; (**d**) Packet Size = 240B; (**e**) Packet Size = 300B.

Similarly, the influence of varying fragment size has been studied for the high priority traffic. The results for delay have been illustrated for each packet size (60B, 120B, 180B, 240B & 300B) in Figure 15. As compared to the results illustrated in Figure 14, those in Figure 15 depict opposite trend. This is because as the fragment size of *T*_1_ traffic would increase, *T*_2_ will face higher delays due to getting a lower chance to interrupt and having to wait for longer. Hence, it has been seen that for each packet size, the delay keeps on increasing for *T*_1_ and decreasing for *T*_2_, as the fragment size increases. Therefore, the fragment size should be chosen efficiently to improve the performance of MAC protocol for prioritized traffic scenarios.

## 7. Conclusions and Future Work

This paper has presented a high-level design and simulation results for a MAC scheme called FROG-MAC, which focuses on providing pre-emptive priority channel access to emergency data in WSNs. Existing protocols could not provide the emergency or high priority data with preemptive priority due to the design limitations. In contrast, FROG-MAC is proposed to offer the high priority traffic with a chance of interrupting ongoing transmission of low priority traffic (using fragmentation). Therefore, FROG-MAC is expected to improve WSN performance for the applications requiring efficiency in terms of delay and reliability. MATLAB simulations show that by increasing the packet size, the delay and PDR both increase for both types of traffic, however the values were found to be significantly lower for the high priority traffic when fragmentation was applied. In addition, it has been observed that decreasing the fragment size of low priority traffic, high priority traffic becomes able to access the medium earlier, and hence its delay reduces. In future, we plan to fully develop a fragmentation-based MAC protocol FROG-MAC including all details such as CSMA, collision detection & avoidance, multi-hop transmissions etc.

## Figures and Tables

**Table 1:** Review of Priority MAC Mechanisms for WSNs.

Protocol	Method	Advantages	Gaps
TP-CAT [24]	Traffic is categorized to have low and high threshold in addition to the priority. Algorithms are proposed to deal with the channel access delay and user conflicts	Channel access delay is greatly reduced for critical data for health monitoring applications. Randomness of back-off counter is ensured.	Critical data cannot interrupt the ongoing transmission.
DPCA [25]	Capacity of routing path and network is measured to ensure transmission of higher priority data to the sink	Higher priority data is transmitted early instead of low priority. Efficient resource utilization.	Complexity lies in measuring capacity of each routing path. Pre-emptive priority is not assigned.
TAP-MAC [5]	Adaptive super-frame structure to provide differentiated channel access to normal and emergency traffic	Emergency traffic gets early channel access due to having shorter contention window.	Contention window is partially shared between regular and emergency traffic which reduces the probability of channel access for high priority traffic. No mechanism has been suggested for interruption of ongoing transmission of regular traffic.
Urg Mac [15]	Integrated mechanisms of duty-cycle reduction, contention window optimization, data rate adjustment and multimedia message passing	Early channel access support for urgent multimedia traffic.	High complexity due to integrating many prioritization schemes. Urgent traffic cannot interrupt ongoing transmission of non-real-time data on the channel and hence the delay would increase.
Priority guaranteed MAC [26]	Modified structure of 802.15.4 integrated with prioritized CSMA/CA and wakeup radio.	Energy efficient transmission of high priority data with guarantee on low latency.	No support for interrupting ongoing transmission. Reliance on wakeup radio which is not yet widely deployed.
PFRC [27]	Data rate adjustment based on the node priority, fairness of throughput and network congestion	All the parent and child nodes can have fair access to the network throughput. Nodes near the sink have higher priority which ensures that data in transit reaches the sink quickly.	No possibility for the nodes to interrupt ongoing transmission. In case the nodes far from the sink generate real-time traffic, they would not be offered high priority.
PA-MAC [21]	Prioritized assignment of contention window and transmission slots to different categories of traffic	Prioritized data has a higher chance of early transmission.	Emergency data cannot interrupt the ongoing transmission. Transmission slots are shared between low and high priority data, lowering the channel access probability for high priority.
Priority-Based Data Gathering Framework [28]	Different values of contention window are assigned to nodes based on their priority.	Nodes with high priority data could transmit earlier. Routing algorithm is also developed to support the priority mechanism.	Interruption of ongoing transmission is not possible, which could cause delay for the delivery of high priority data.
I-MAC [29]	Modified super frame structure with short interruption slots included	The coordinator can interrupt the executing super frame during the interruption slot in order to provide the channel access to the node with urgent data.	Centralized scheme requiring the nodes with urgent data to wait for the interruption by coordinator.
PATS [30]	Game theory algorithm is used for assigning priority to the nodes with critical health monitoring data in WBAN.	The critical health monitoring data could be transmitted earlier due to efficient priority assignment.	Complex algorithm has been used which might not be fully/efficiently deployed on the WSN nodes with limited computing capability. No support for interruption of low priority regular health monitoring data by the emergency traffic.
RushNet [8]	High priority data is transferred using low overhead mechanism through channel capture and power difference effects.	Reduced overhead due to no need for explicit coordination between the nodes. Allows transmission of high priority data even when the channel is busy.	Requires two transceivers. Efficient signal processing is required at the receiver to be able to differentiate between the high & low priority traffic.

**Table 2:** Experimental Settings.

Simulation Parameters	Value
Simulation Duration	6000 s
Number of iterations for each result	50
Transmission Bit Rate	20 kbps
Number of nodes	100
Priority levels	2
Message generation interval for *T*_1_	60 s
Message generation interval for *T*_2_	120 s (Mean of the exponential distribution)
Packet Size (for both types)	60B, 120B, 180B, 240B, 300B
Time taken to transmit each byte (Packetization delay)	0.4 ms
Interruptible Period *T_INT_*	0.4 ms
Checking Period *T_CH_*	Random value (<0.4 ms)
